# New multiplex LC-MS/MS method for lipid biomarker analysis of inherited neurodegenerative metabolic diseases

**DOI:** 10.1016/j.jlr.2025.100967

**Published:** 2025-12-20

**Authors:** Anna Sidorina, Giulio Catesini, Federica Deodato, Sara Boenzi, Diego Martinelli, Cristiano Rizzo, Carlo Dionisi-Vici

**Affiliations:** Division of Metabolic Diseases and Hepatology, Bambino Gesù Childrens Hospital IRCCS, Rome, Italy

**Keywords:** biomarker analysis, brain lipids, cerebrosides, ceramides, glycolipids, lysophospholipid, sphingolipids, neurodegenerative metabolic diseases, newborn screening, MS/MS

## Abstract

A significant number of inherited neurodegenerative metabolic diseases (NMDs) arise from altered lipid metabolism, including impaired degradation of sphingolipids and dysfunction in organelle-related machineries involved in lipid processing and trafficking. These lipid dysregulations profoundly impact cellular membranes, signaling pathways, and myelin integrity, contributing to the complex and multisystemic clinical phenotypes characteristic of NMD, which often complicate diagnosis and delay treatment initiation. Here, we present a high-throughput, multiplex LC-MS/MS method for the analysis of an extended panel of NMD biomarkers in plasma and dried blood spots. One-step sample extraction and targeted LC-MS/MS acquisitions in positive and negative ionization allowed the simultaneous measurement of 13 diagnostic biomarkers associated with GM1 and GM2 gangliosidosis, Fabry, Gaucher, and Krabbe diseases, acid sphingomyelinase deficiency, Niemann-Pick disease type C, X-linked adrenoleukodystrophy, peroxisomal biogenesis disorders (Zellweger syndrome), metachromatic leukodystrophy, and mental retardation, enteropathy, deafness, neuropathy, ichthyosis, keratoderma (MEDNIK)/MEDNIK-like syndromes, a disorder of cellular trafficking. The method was analytically and clinically validated, confirming the diagnosis of all targeted NMDs in samples from 89 patients. Additionally, the method allowed the differentiation of X-linked adrenoleukodystrophy from peroxisomal biogenesis disorder and revealed the elevation of C18- and C16-sulfatides in Krabbe disease and MEDNIK syndrome, respectively. This multiplex assay enhances diagnostic efficiency and expands the discovery of novel biomarkers, enabling the quantification of diagnostic markers for a wide range of NMDs. The method is suitable for diagnosis of NMD, as a first- or second-tier test in neonatal screening, as confirmatory testing of variant of unknown significance in genetic panels and for longitudinal monitoring in treatable diseases.

Inherited metabolic disorders (IMDs) are genetic conditions that alter metabolic cellular pathways, resulting in a large spectrum of clinical manifestations that affect multiple organs and systems. IMDs are individually rare but collectively numerous, and a recently proposed nosology of IMD included 1,450 disorders (1). Those with a major involvement of the central nervous system can be defined as “neurodegenerative metabolic diseases” (NMDs), and an increasing number are or are becoming treatable with enzyme replacement therapies, hematopoietic stem cell transplantation (HSCT), specific drugs, and gene therapy (2, 3). According to IMD classification, NMD refers to the categories of complex molecules and sphingolipid degradation, or to disorders of lipid metabolism and organelle biogenesis, dynamics and interactions, which play a critical role in cell structure, signaling, redox and energy homeostasis (1). Age at onset is variable from early infancy to adulthood, and patients present with progressive neurological deterioration, motor dysfunctions, epilepsy, neuro-ophthalmological signs, acute neurological events, hearing impairment, intellectual disabilities, psychiatric symptoms, and abnormal MRI findings. Depending on the individual disease, neurological signs are associated with specific organ signs affecting the liver, lung, heart, kidney, endocrine, and hematopoietic systems (4). However, the complexity and heterogeneity of clinical phenotypes represents a major challenge and often causes delayed identification. The gold standard for NMD diagnosis relies on a combined approach based on biochemical and genetic tests, which allows a precise disease identification (5). Biochemical tests include the measurement of enzyme activity and/or the analysis of specific biomarkers in biological fluids. To fulfil this need and to improve diagnostic capacities, LC-MS/MS may allow the detection of biomarkers in biological samples (6, 7, 8, 9, 10, 11). More recently, to allow an early treatment initiation, an increasing number of NMDs have been included in newborn screening (NBS) panels (12). NBS is carried out by LC-MS/MS in dried blood spot (DBS) by measuring the enzyme activity or by identifying disease-associated biomarkers (12). However, the diagnosis of some NMDs may be hampered by pseudodeficiency alleles, which lower the enzymatic activity to a disease range in normal individuals, thus complicating the diagnostic process and NBS programs (13).

Several LC-MS/MS methods are focused on biomarker analyses for single disease, such as glucosylsfingosine (LysoGb1) for Gaucher disease, lyso-globotriaosylsphingosine (LysoGB3) for Fabry disease, 1-hexacosanoyl-2-hydroxy-*sn*-glycero-3-phosphocholine (LPC26:0) for X-linked adrenoleukodystrophy (X-ALD), sulfatides for metachromatic leukodystrophy (MLD), lyso-monosialoganglioside GM1 (LysoGM1) for GM1-gangliosidosis, and galactosylsphingosine for Krabbe disease (14, 15, 16, 17, 18, 19, 20, 21, 22, 23, 24). To improve diagnostic performance, multiplex methods providing the simultaneous measurements of selected lysosphingolipids, glycosphingolipids, oligosaccharides, and mucopolysaccharides have been developed (6, 7, 9, 11, 25). However, these methods do not allow the simultaneous analysis of sulfatides and LPC26:0, the biomarkers of MLD, X-ALD, and peroxisomal disorders, recently proposed in NBS programs. To this purpose, NBS algorithms have included first- and second-tier testing combining enzymatic activity assays and biomarker analysis. In this setting, a high-throughput multiplex LC-MS/MS assay combining enzymatic activity with biomarker analysis was designed to screen 18 disorders, including 15 lysosomal storage diseases, X-ALD, galactosemia due to galactose-1-phosphate uridyl transferase deficiency and biotinidase deficiency (8).

In this article, we introduce a high-throughput multiplex LC-MS/MS method for the simultaneous detection in plasma and DBS of an extended panel of NMD biomarkers, which includes Fabry, Gaucher, and infantile Krabbe diseases, acid sphingomyelinase deficiency (ASMD), Niemann-Pick disease type C (NPC), GM1-and GM2-gangliosidoses, MLD, X-ALD, peroxisomal biogenesis disorders (PBDs) and of MEDNIK (mental retardation, enteropathy, deafness, neuropathy, ichthyosis, keratoderma) and MEDNIK-like syndromes—two ultrarare disorders of cellular trafficking characterized by a complex neurocutaneous phenotype and affecting copper and very long-chain fatty acid metabolism (26, 27). This method allows the rapid diagnosis of 12 genetically different NMDs, several of which is treatable or have ongoing new therapies.

## Materials and Methods

### Chemicals and reagents

LysoGM1, LysoGB3, galactosylsphingosine, and lyso-sphingomyelin (LysoSM) were purchased from Sigma-Aldrich/MERCK (Burlington, MA). Galactosylsphingosine was used as the reference standard also for glucosylsphingosine, and both were named as lyso-hexosylsphingosine (LysoHexSph). 1-LPC26:0 was purchased from Avanti Polar Lipids (Alabaster, AL). Lyso-monosialoganglioside GM2 (LysoGM2) NH4+ salt, *N*-hexadecanoyl-sulfatide (C16-sulfatide), and *N*-octadecanoyl-sulfatide (C18-sulfatide) were bought from Matreya LLC (State College, PA). 3α,7α-Dihydroxy-5β-cholestan-26-oic acid (DHCA) and 3α,7α,12α-trihydroxy-5β-cholestan-26-oic acid (THCA) were purchased from VU Medical Center Metabolic Laboratory (Amsterdam, The Netherlands). Stable isotope-labeled internal standards (ISs) included lyso-sphingomyelin-D7 (LysoSM-D7; Sigma-Aldrich/MERCK, Burlington, MA), 1-hexacosanoyl-d4-2-hydroxy-*sn*-glycero-3-phosphocholine (Avanti Polar Lipids, Alabaster, AL), N-omega-CD3-octadecanoyl-sulfatide (C18-D3-sulfatide; Matreya LLC, State College, PA), 27,27,27[D3]-3α,7α-dihydroxy-5β-cholestan-26-oic acid (DHCA-D3, VU Medical Center Metabolic Laboratory, Amsterdam, The Netherlands), 27,27,27-[D3]-3α,7α,12α-trihydroxy-5β-cholestan-26-oic acid (THCA-D3; VU Medical Center Metabolic Laboratory (Amsterdam, The Netherlands).

Acetonitrile, methanol (HPLC-MS-gradient grade) and chloroform were purchased from Sigma-Aldrich (St Louis, MO). The ULC-MS-grade 99% formic acid was supplied by Biosolve Chimie (Dieuze, France). The laboratory reagent grade acetone was purchased from Fisher Scientific UK Ltd (Loughborough, UK). Ultrapure water was generated using the Milli-Q system (Millipore, Bedford, MA). The stock standard solutions for all analytes were prepared by solving the powder materials in a mix of methanol and chloroform as recommended by the manufacturers.

### Patient samples

Stored plasma and DBS samples were obtained from 89 NMD patients followed at the Bambino Gesù Children's Hospital (OPBG). Patient’s age varied from 0.1 to 72 years (median, 9.7 years). Control samples were obtained from 122 (plasma) and 188 (DBS) anonymized sex- and age-matched individuals. Patient samples included 9 GM1-gangliosidosis (MIM: 611458; 0.7–19.8 years), 4 GM2-gangliosidosis (MIM: 606869; 1.8–6.0 years) including two Sandhoff (MIM: 268800) and two Tay-Sachs (MIM: 272800), two naive male Fabry (MIM: 300644; 9.7–10.1 years), one symptomatic naive female Fabry and two asymptomatic female-carriers (2.1–13.6 years), two naive Gaucher disease (MIM: 606463; 0.1–15.3 years) including one type 1 (MIM: 230800) and one type 2 (MIM: 230900), four infantile Krabbe disease (MIM: 606890; 0.1–0.9 years), one of whom was then treated by HSCT (age at sampling = 0.8–4.1 years), six ASMDs (MIM: 607608; 0.5–17.8 years), 16 NPCs (MIM: 607623; 0.3–28.2 years), four lysosomal acid lipase (LAL) deficiency (MIM: 613497; 0.5–14.8 years), eight male X-ALDs (MIM: 300371; 4.2–57.2 years), 11 with adrenomyeloneuropathy (AMN) (MIM: 300371; 34.7–63.2 years), five X-ALD female carriers (36.7–71.8 years), six PBDs (MIM: 601498, 603360; 0.1–20.4 years), one late-infantile MLD (MIM: 607574; 2.8–3.1 years), three early-juvenile MLDs (MIM: 607574; 5.8–13.2 years), one MEDNIK and three MEDNIK-like syndromes (MIM: 603531, 600157; 1.2–15.8 years). All patients enrolled had a confirmed diagnosis by enzyme and/or molecular analysis. Human EDTA blood was centrifugated, and the separated plasma was stored at −80°C until analysis. DBS samples were stored at −20°C after collection. The experimental protocol was reviewed and approved by the OPBG Ethical Committee (2119_OPBG_2020). The study was performed in accordance with the Declaration of Helsinki, and informed consents were obtained from all participants/parents.

#### Calibration standards and quality control preparation

##### Plasma

The IS solution was prepared as a mix of LysoSM-D7 (50 nM), LPC26-D4 (1 μM), C18-D3-sulfatide (1 μM), DHCA-D3 (5 μM), and THCA-D3 (5 μM) in methanol. The extraction solution, functioning also as plasma protein precipitant, was composed of methanol, acetone, and water (60:30:10). Seven-point calibration curves were prepared by spiking the pure standards with pooled human plasma with following concentration ranges: 0–200 nM for LysoGb3; 0–1,000 nM for LysoGM1, LysoGM2, LysoSM, LysoHexSph, and C18-sulfatide; and 0–2,000 nM for LPC26:0 and C16-sulfatide. Aliquots of standards in methanol were placed in a glass tube and dried under a nitrogen stream, then a corresponding amount of plasma was added to obtain the highest concentration point of the calibration curve. The glass tube with plasma was mixed on a thermomixer for over 23 h at 37°C and 450 rpm to achieve a homogeneous and complete solubilization of standards. Afterward, the dilution with pooled plasma was made to obtain other calibration points.

##### Dried Blood Spots

The IS solution, functioning also as an extraction solution, was prepared as a mix of LysoSM-D7 (2.5 nM), 1-hexacosanoyl-d4-2-hydroxy-*sn*-glycero-3-phosphocholine (50 nM), C18-D3-sulfatide (50 nM), DHCA-D3 (500 nM), and THCA-D3 (500 nM) in methanol. Seven-point calibration curves were prepared with the following concentration ranges: 0–500 nM for LysoGb3 and LysoSM; 0–1,000 nM for LysoGM1, LysoGM2, LysoHexSph, and C18-sulfatide; 0–2,000 nM for LPC26:0 and C16-sulfatide. The DBS with known concentrations for calibration curve and quality controls (QCs) were prepared as follows: 50 μl of human blood was dropped onto the DBS collection card to fill the printed 13-mm diameter circle form and left for complete drying (about 3 h on air); after that, 25 μl of a standard solution mix was dropped on DBS and left on air for complete methanol evaporation. The used 25 μl of methanol solution was sufficient to fill completely the DBS area of the 13 mm. The resulting standard concentration in spiked DBS was assumed as 2-fold reduced original concentration in the methanol solution.

The precision and accuracy of the assay were evaluated using the QCs at three concentration levels, which were prepared from pooled plasma or blood added with pure standards (Supplemental Tables S1, S2).

The concentrations of *N*-palmitoyl-O-phosphocholineserine (Lyso509), of C16-OH- and C16:1-OH-sulfatides were expressed as multiple of median (MOM), through the estimation of the peak area ratios between analyzed samples and control samples. The indicative concentrations of DHCA and THCA were estimated as ([analyte peak area/IS area] ∗ IS concentration). Assay of interday/intraday variability of DHCA and THCA concentrations was carried out by measuring the endogenous concentrations in the samples from a PBD patient (labeled as QC1).

To evaluate the influence of temperature and store conditions on the stability of analytes, plasma QCs were retested after three freeze–thaw cycles. For DBS, the comparison of analyte concentrations was made between samples kept at room temperature and at −20°C for 6 months.

#### Sample preparation

##### Plasma

Plasma samples (50 μl) were placed into a 1.5 ml collection tube containing 10 μl of IS solution mix. To extract all analytes and precipitate plasma proteins, 500 μl of extraction solution were then added. After 5 s mixing, the sample was sonicated in a bath for 6 min and centrifuged for 9 min at 13.000 rpm. The supernatant solution was transferred in an HPLC glass vial and evaporated under a nitrogen stream. To reconstitute the sample, 100 μl of pure methanol were added and, after 2 s mixing, all samples were placed in HPLC glass vials.

##### Dried Blood Spot

A 3.2 mm DBS punch was placed into a 96-well plate, and 200 μl of IS solution in methanol was added. The sealed plate was incubated on a thermomixer at 40°C at 400 rpm for 1 h, and the methanol extract was then transferred into a glass vial for injection.

#### LC-MS/MS

Analysis of all species was performed using LC-MS/MS on ExionLC™ and QTRAP 6500^+^ system (AB Sciex LLC, Framingham, MA). The reverse-phase chromatography was carried out on a Gemini C6-Phenyl 100 × 5 mm column with a 3 μm particle size (Phenomenex, Torrance, CA). Mobile phases were composed of water and 40% acetonitrile (phase A), 95% acetonitrile (phase B), 10 mM ammonium formate, and 0.1% formic acid. The chromatographic gradient was defined as shown in Supplemental Table S3.

MS detection was conducted both in positive and negative modes using an ESI by switching polarity in the same run. The source temperature was set at 550°C; curtain gas—20; ion source gas1—45; ion source gas2—45; and collision gas—high. We chose a scheduled acquisition mode to increase the sensitivity and simplify the view of chromatograms, excluding isobar species. Supplemental Table S4 contains the list of multiple reaction monitoring transitions for all analytes and MS settings.

The technical features described in this section are complete to carry out the method and are included in a patent application concerning a multiplex LC-MS/MS method for the simultaneous detection in plasma and DBS of an extended panel of NMD biomarkers (28).

#### Statistical analysis

Statistical differences between mean concentrations in control and patient populations and correlations between patients' age and biomarker levels were evaluated by nonparametric tests (Mann-Whitney *U* test, Spearman's correlation coefficients) on GraphPad Prism 10 (GraphPad Software, Inc). Statistical significance was evaluated only between groups with 2 or more observations, with the indication of median and 25th to 75th percentile on bar graphs.

## Results

**Fig. 1:**
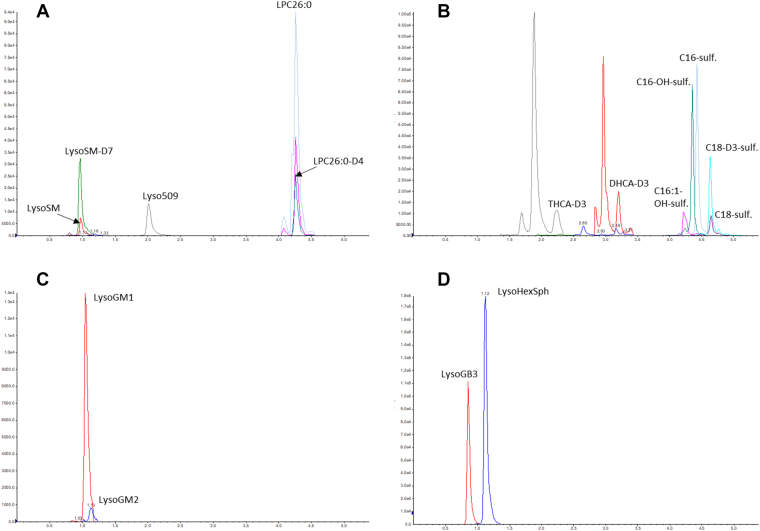
Extracted ion chromatograms from a plasma sample (A, B) and a methanol solution spiked with LysoGM1, LysoGM2 (C), LysoGB3, and LysoHexSph (D). Transitions acquired in positive ionization mode (A, C, and D); transitions acquired in negative ionization mode (B).

Figure 1 shows the extracted ion chromatograms representative of all analytes in positive and negative ionization. As can be seen in Fig. 1, sufficient chromatographic resolution was obtained for all analytes. It is important to note that the ISs DHCA-D3 and THCA-D3 have isobaric endogenous compounds present in plasma, which appear as two additional peaks preceding the target peak. A total ion current from a plasma sample is shown in Supplemental Fig. S1.

### Method validation

Calibration curves in plasma and DBS for analytes with available commercial standards (LysoGM1, LysoGM2, LysoGB3, LysoHexSph, LysoSM, LPC26:0, C18-sulfatide, and C16-sulfatide) are shown in Supplemental Figs. S2, S3. The correlation coefficients (*R*^2^) referring to linear dependence between analyte concentration and measured signal were all ≥0.99, both in plasma and DBS. The accuracy of the method was assessed by performing recovery studies using the QCs. Recovery (in %) was calculated as the mean of the analyzed amount divided by the theoretical amount and multiplied by 100. The best recoveries were obtained for LysoGM1, LysoGM2, LysoGB3, LysoSM, and LysoHexSph in plasma and for C16- and C18-sulfatides in DBS. The relative errors in accuracy measurements were <22% for all analytes, both in plasma and DBS.

Within-run and between-run precision were determined by preparing and analyzing each QC five times per run, over 3 consecutive days. For DBS analysis, the interday assay was made utilizing punches both from the center and sides of the spot to account for possible chromatographic effects of filter paper on analytes.

The CVs for all analytes in plasma were ≤20%. DBS samples had greater variability, reaching 24% in interday measurements for LysoSM and LPC26:0% and 18% to 28% for LysoGM2. The results of accuracy and precision evaluation for plasma and DBS samples are shown in Supplemental Tables S5, S6.

The limit of detection (LOD) and limit of quantification (LOQ) for each biomarker were determined using a signal-to-noise ratio of 3 for LOD and of 10 for LOQ (Supplemental Table S7). No significant changes in analyte concentrations were observed after three freeze-thaw cycles in plasma samples or in DBS kept for 6 months at room temperature or at −20°C.

### Quantification of biomarkers and clinical validation

Plasma and DBS levels of biomarkers in controls and patients are given in Supplemental Tables S8, S9.

LysoGM1 and LysoGM2 were undetectable in plasma and/or in DBS of the control population and in samples from patients with nontarget diseases. For LysoGB3 and LysoHexSph, detectable at very low concentrations in control DBS samples, we considered values >LOD, but <LOQ, for indicative evaluation. Quantification of the two bile acids, DHCA and THCA, was possible only in plasma samples of PBD patients (Supplemental Table S10).

**Fig. 2:**
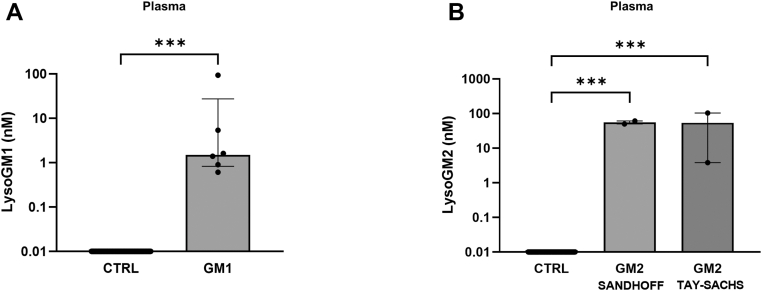
Plasma concentrations of LysoGM1 in patients with GM1 gangliosidosis (A); plasma concentrations of LysoGM2 in patients with GM2 gangliosidosis, Sandhoff, and Tay-Sachs variants (B).

LysoGM1 levels were elevated in all plasma of GM1 gangliosidosis patients (0.6–93.5 nM), showing a strong negative correlation with patients' age (*r* = −0.95, *P* < 0.0004) (Fig. 2A). In DBS, LysoGM1 was detectable only in one of five samples obtained from the patient showing the highest LysoGM1 concentration in plasma.

As shown in Fig. 2B, elevation of LysoGM2 levels was seen in all plasma samples from patients with GM2 gangliosidosis: two with Tay-Sachs (3.8–104 nM) and two with Sandhoff disease (49.9–60.9 nM), whereas GM2 levels in DBS were not detectable.

**Fig. 3:**
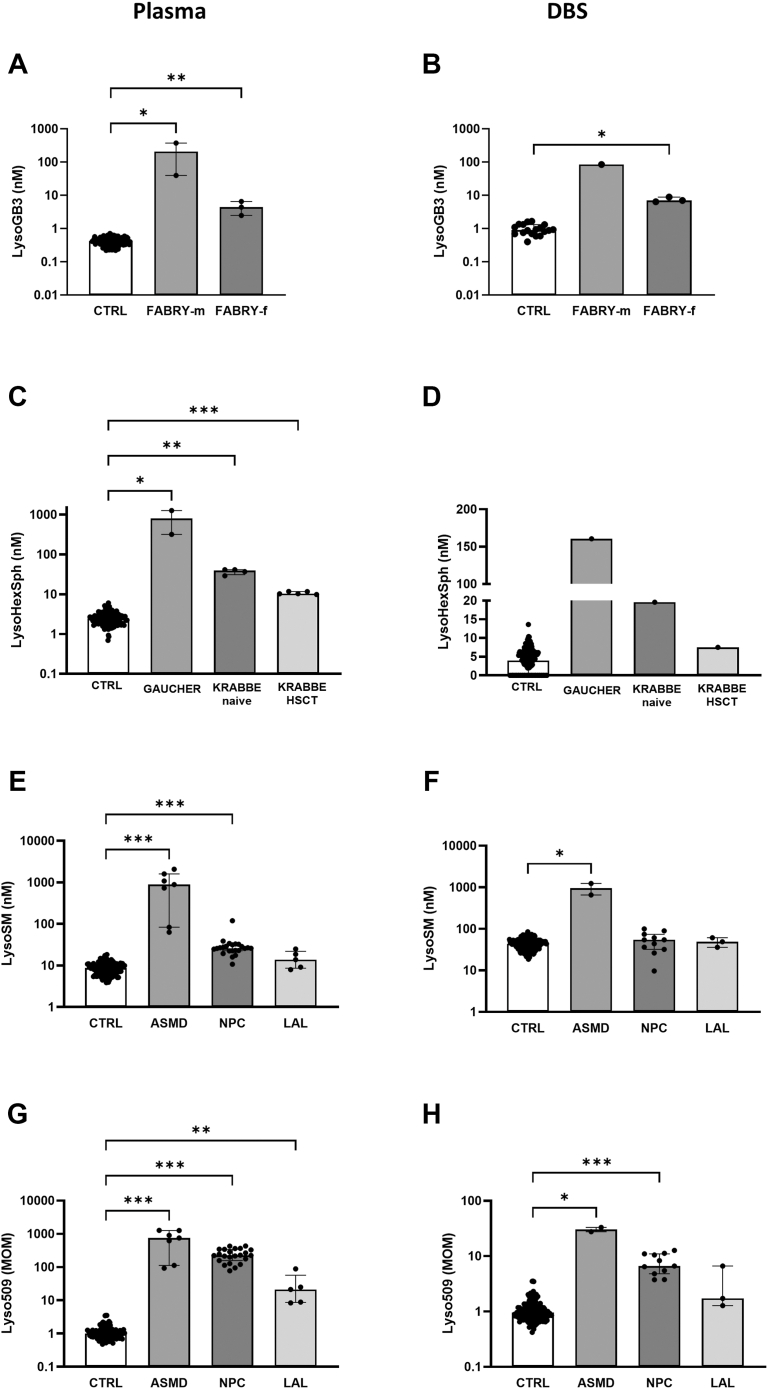
Plasma (left panels) and DBS (right panels) concentrations of LysoGB3 in male patients with Fabry disease (FABRY-m) and in heterozygous female carriers (FABRY-f) (A, B). Plasma and DBS concentrations of LysoHexSph in patients with Gaucher disease and Krabbe disease (C, D). Plasma and DBS concentrations of LysoSM (E, F) and Lyso509 (G, H) in patients with ASMD, NPC, and LAL. ∗∗∗*P* < 0.001, ∗∗*P* < 0.01, ∗*P* < 0.05, NS, nonsignificant. Statistical significance was calculated only between groups with ≥3 samples.

Levels of LysoGB3 were clearly elevated in plasma and DBS samples from naive Fabry male patients (39.5–373 nM in plasma and 84.7 nM in DBS) compared with the naive symptomatic Fabry female patient and female carriers (2.5–6.5 nM in plasma and 6.4–8.9 nM in DBS, Fig. 3A, B) and easily distinguished patients from negative controls. A slight increase of LysoGB3, exceeding control values, was seen in plasma, but not in DBS, from Gaucher patients with higher levels in subtype 2 (Supplemental Tables S8, S9).

Our method cannot separate the two isomers glucosylsphingosine and galactosylsphingosine; therefore, LysoHexSph, which comprised both compounds, was elevated in plasma and DBS samples from Gaucher (317 nM in type 1 and 1,263 nM in type 2 in plasma, 160.5 nM in type 1 in DBS) and from untreated infantile Krabbe patients (28.9–41.1 nM in plasma and 19.6 nM in DBS), with higher levels in Gaucher disease regardless disease subtypes (Fig. 3C, D, Supplemental Tables S8, S9).

Repeated determinations of LysoHexSph in plasma and DBS in the single infantile Krabbe patient, treated by HSCT, displayed lower levels compared with the 4 untreated patients (10.3 nM vs. 39 nM in plasma and 7.5 nM vs. 19.6 nM in DBS).

Plasma LysoSM was significantly elevated both in ASMD (63–2,088 nM) and in NPC (10.8–120 nM) patients, with higher levels detected in ASMD (Fig. 3E, Supplemental Table S8). Plasma LysoSM was also mildly increased in LAL and type 1 Gaucher patients (Supplemental Table S8). In DBS samples, LysoSM was markedly elevated only in ASMD (652–1,232 nM), easily distinguishing patients from negative controls (Fig. 3F).

Plasma levels of Lyso509 were significantly elevated in ASMD (93–1281 MOM), NPC (77–432 MOM), and LAL (8.4–89.1 MOM) patients compared with controls (0.5–3.5 MOM) (Fig. 3G, Supplemental Table S8). Milder increase of plasma Lyso509 was also seen in 2/6 GM1-gangliosidosis patients (4.6–8.6 MOM), in two-fourths of GM2-gangliosidosis patients (6.0–10.1 MOM), in half of Fabry male patients (7.3 MOM), and in 2/2 Gaucher patients (4.6 MOM in type 2 and 12.0 MOM in type 1) (Supplemental Table S8). In DBS, Lyso509 was increased in ASMD (27.7–33.0 MOM), clearly distinguishing patients from negative controls (0.4–2.8 MOM) (Fig. 3H). Although being significantly elevated also in NPC, in some patients’ samples, levels overlapped those of controls. Similarly, only one of three LAL patients had elevated DBS Lyso509 levels, whereas in two patients, they were within control range (Fig. 3H). Mild DBS elevation of Lyso509 was also seen in two-fifths of GM1-gangliosidosis patients (5.5–8.6 MOM), in one-fourth of juvenile MLD patients (6.2 MOM), and in 2/6 PBD patients (4.2–7.2 MOM) (Supplemental Table S9).

**Fig. 4:**
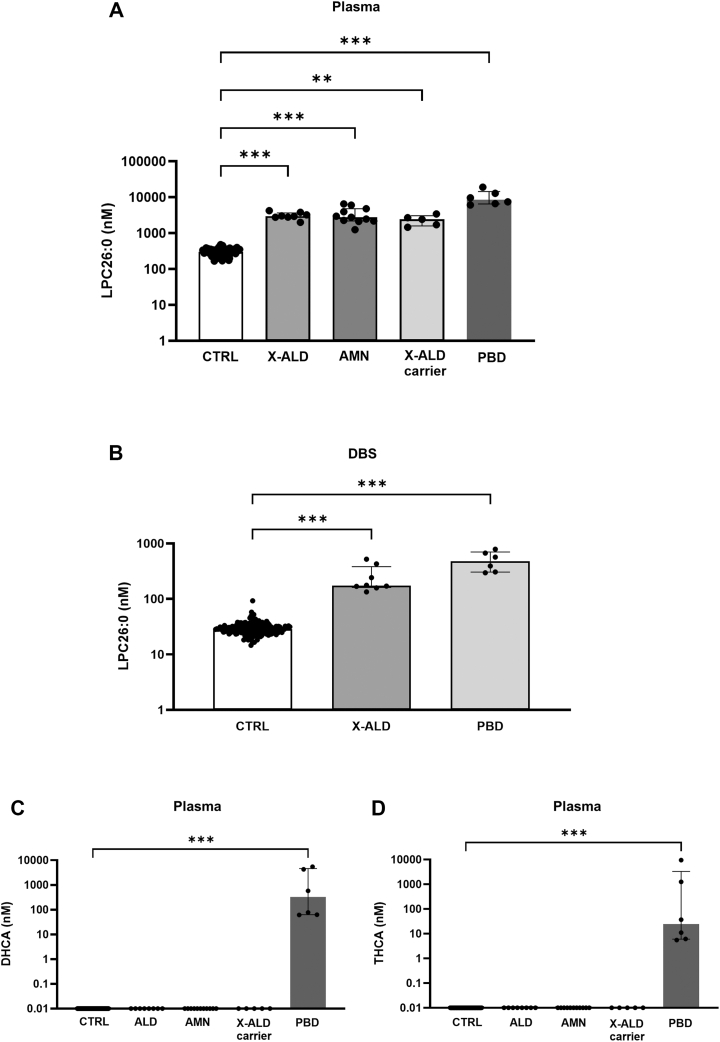
Plasma concentrations of LPC26:0 in plasma (A) and DBS (B) in patients with X-ALD, AMN, in heterozygous female X-ALD carriers, and in patients with PBD. Plasma concentrations of bile acids DHCA (C) and THCA (D) in patients with PBD. ∗∗∗*P* < 0.001, ∗∗*P* < 0.01, ∗*P* < 0.05, NS, nonsignificant.

LPC26:0 was significantly elevated in all plasma samples from male X-ALD patients (2,013–4,248 nM), male AMN patients (1,253–6,548 nM), female X-ALD carriers (1,463–3,433 nM), and PBD patients (6,098–19,098 nM), with the highest values in PBD (Fig. 4A, Supplemental Table S8). The concentrations of LPC26:0 were significantly elevated in DBS from X-ALD (134–522 nM) and PBD (296–788 nM) patients, with the highest values in the PBD group (Fig. 4B, Supplemental Table S9). No DBS samples were available from AMN patients and female X-ALD carriers.

Elevated levels of the bile acids DHCA and THCA were seen only in plasma samples from PBD patients, with undetectable (<LOD) levels in ALD and ANM patients, in female ALD carriers, and in control samples (Fig. 4C, D) and in patients with other nonrelated diseases (Supplemental Table S10). The levels of DHCA and THCA were <LOD in all DBS samples, including those obtained from PBD patients.

**Fig. 5:**
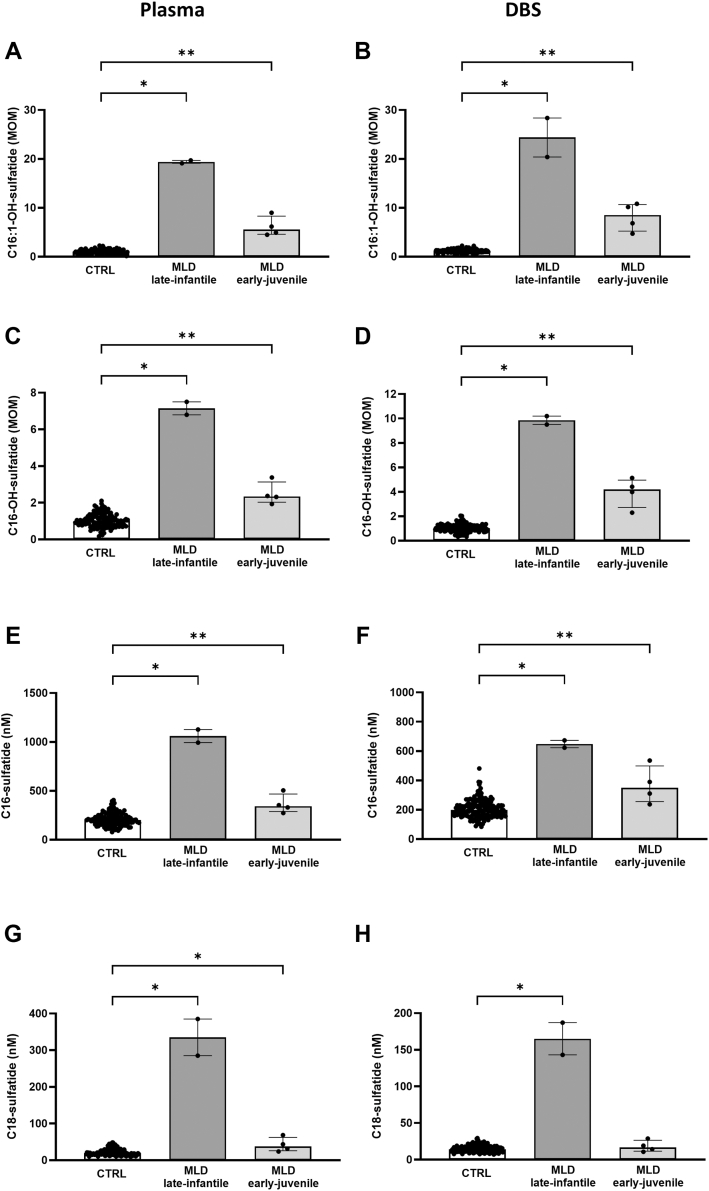
Plasma (left panels) and DBS (right panels) concentrations of the four sulfatides C16:1-OH (A, B), C16-OH (C, D), C16 (E, F), and C18 (G, H) in patients with MLD. Late-infantile subtype, MLD late-infantile; early-juvenile subtype, MLD early-juvenile. ∗∗∗*P* < 0.001, ∗∗*P* < 0.01, ∗*P* < 0.05, NS, nonsignificant. Statistical significance was calculated only between groups with ≥3 samples.

Figure 5 shows the concentration of the four sulfatides measured in plasma and DBS. All were significantly elevated in MLD patients, with the C16:1-OH-sulfatide being the most discriminant biomarker, with no overlapping values between patients and controls, both in plasma and in DBS (Fig. 5A, B). MLD patient with the late-infantile disease subtype showed elevated plasma and levels of all sulfatide species, compared with those with the early-juvenile subtype, who presented only a significant increase of C16:1-OH- and C16-OH-sulfatides, with mild or absent elevation of C18- and C16-sulfatides (Supplemental Tables S8, S9).

**Fig. 6:**
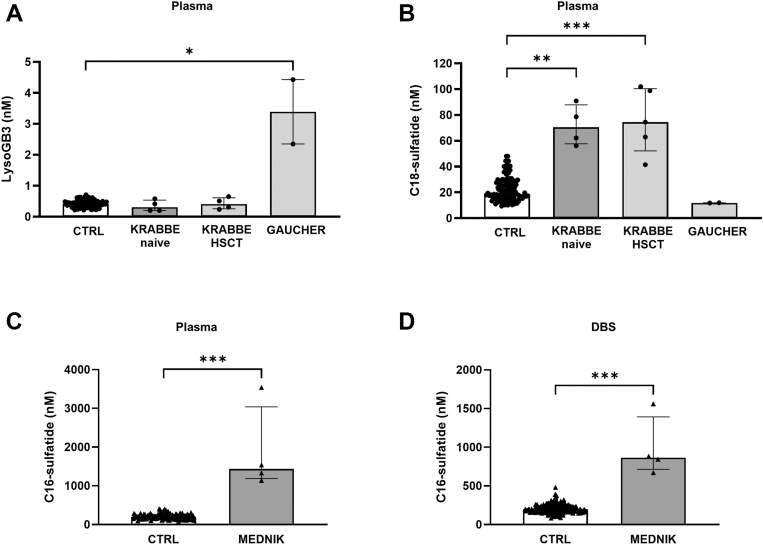
Novel proposed biomarkers. Plasma concentrations of LysoGB3 and of C18-sulfatide in patients with Krabbe disease and Gaucher disease (A, B). Plasma (C) and DBS (D) concentrations of C16-sulfatide in patients with MEDNIK and MEDNIK-like syndrome. ∗∗∗*P* < 0.001, ∗∗*P* < 0.01, NS, nonsignificant. Statistical significance was calculated only between groups with ≥3 samples.

Additional biomarkers, indicative of some diseases, are presented in Fig. 6A–C. LysoGB3 was significantly increased in Gaucher patients, whereas all infantile Krabbe patients were characterized by elevated C18-sulfatide with no difference between naive and the single HSCT-treated one (Fig. 6A, B). C16-sulfatide was massively elevated in plasma and DBS samples from patients with MEDNIK and MEDNIK-like syndrome (1,139–3,537 nM in plasma and 672–1,562 nM in DBS, Fig. 6C, D, Supplemental Tables S8, S9).

## Discussion

This new multiplex LC-MS/MS method, designed for the analysis of a large number of biomarkers, represents a high-throughput analytical tool, suitable for diagnosis and screening of NMD, and for treatment monitoring in diseases with available therapies. The method was validated in plasma and DBS, providing good accuracy, precision, and reproducibility for quantitatively measurable analytes. The semiquantitative assay of metabolites through MOM or isotopic dilution also provided effective biomarker discrimination. The clinical validation of the method applied to a large number of NMD demonstrated high diagnostic specificity, providing physiological and pathological concentration ranges in plasma and, for the majority of biomarkers, in DBS. Some limitations have been encountered for the measurement in DBS of few analytes not included in targeted NBS panels (i.e., lysoGM1, LysoGM2, and bile acids), due to a very low signal intensity, as the consequence of the smaller volume (3 μl of DBS vs. 50 μl of plasma), of a stronger matrix effect in DBS extract, or to poorer ionization.

Plasma LysoGM1 and LysoGM2 were elevated in patients with GM1- and GM2-gangliosidosis, respectively, with no difference between Tay-Sachs and Sandhoff diseases. LysoGM1 concentrations demonstrated a strong negative correlation with patients' age, consistent with a more severe disease in young early-onset patients (29).

LysogGB3 was increased in Fabry patients, with higher plasma and DBS levels in male patients compared with the heterozygous females, two asymptomatic and one symptomatic on enzyme replacement therapies. Gaucher patients had also increased LysoGB3; however, the simultaneous measurement of LysoHexSph clearly distinguished Gaucher from Fabry disease.

The method does not differentiate between the two isomers of LysoHexSph, Lyso-glucosylsphingosine (characteristic of Gaucher disease) and Lyso-galactosylsphingosine (characteristic of Krabbe disease). However, the simultaneous multiplex biomarker analysis showed that the two diseases can be easily distinguished. The absolute values of LysoHexSph are significantly higher in Gaucher, particularly in type 2 patients, than in Krabbe disease. LysoGB3 was elevated only in Gaucher disease, whereas C18-sulfatide was elevated only in Krabbe disease.

Simultaneous analysis of LysoSM and Lyso509 allowed a clear differentiation between ASMD and NPC disease. In plasma, the levels of LysoSM were significantly higher in ASMD, whereas the ratio of Lyso509/LysoSM was higher in NPC and not in ASMD. In DBS, both LysoSM and Lyso509 were significantly elevated in ASMD, whereas in NPC samples, only Lyso509 was elevated with normal levels of LysoSM. These results are in accordance with previous studies, showing insufficient capacity of DBS LysoSM and Lyso509 to identify NPC disease and poor correlation between plasma and DBS levels of these biomarkers (7, 30). Similar findings were also true for LAL patients, who demonstrated elevated levels of Lyso509 only in plasma samples but not in DBS. However, it should be noted that elevated Lyso509 levels alone are not sufficient for discrimination of LAL disease, given the poor specificity of this biomarker and its moderate elevation recorded also in some samples from other NMD, such as in GM1 and GM2 gangliosidosis, Fabry, and Gaucher diseases. Similar to our findings, an unspecific elevation of LysoSM and Lyso509 disease was previously reported in Gaucher disease (31).

LPC26:0 is a well-established biomarker of peroxisomal disorders, and its assessment is included in NBS programs for X-ALD screening (32). Recently, it was shown that NBS levels of LPC26:0 had the potential to discriminate ALD patients developing childhood disease–related phenotypes from those without early clinical manifestations (33). In the context of our study, the measurement of LPC26:0 in plasma and DBS permitted the net differentiation of X-ALD, AMN, and PBD patients from controls, with no elevation detectable in patients with other NMDs. The absolute values of LPC26:0 in plasma were significantly elevated in PBD compared with X-ALD and AMN, with some overlapping values. As already reported, plasma LPC26:0 was also elevated in X-ALD carriers overlapping the values in affected male subjects (34, 35, 36). However, the simultaneous analysis of the bile acids, THCA and DHCA, showed the selective elevation in PBD only, allowing a net distinction with X-ALD and AMN. In DBS, the quantification of LPC26:0 did not allow a clear discrimination between PBD and X-ALD, due to the presence of overlapping values between the two conditions. The observed differences between plasma and DBS are in accordance with other studies, where the measurement of LPC26:0 provided concentrations similar to our ranges both in plasma and in DBS (34, 37). Worth noting, we found that the use of LPC26:0 product ion with *m/z* 104 for quantitative analysis gave more reproducible results with respect to the more intense product ion with *m/z* 184 as previously reported (38).

Our method includes the analysis of four sulfatides. Recent NBS pilot studies on MLD successfully demonstrated the high-discriminating rate of 16-OH- and 16:1-OH-sulfatides as biomarkers in DBS (10, 20). Our study confirmed that the two hydroxylated forms of C16-sulfatides were the best discriminant biomarkers for MLD, both in plasma and DBS. The simultaneous analysis of C18- and C16-sulfatides showed that they were variably elevated in MLD, C18-sulfatide was elevated in infantile Krabbe disease, whereas C16-sulfatide was increased in MEDNIK and MEDNIK-like syndrome. Elevated C18-sulfatide, possibly arising from the characteristic severe demyelinating processes, was previously reported in Krabbe disease (17, 39) but indeed, the new multiplex method showed that the combined elevation of C18-sulfatide and LysoHexSph represents a novel peculiar profile in infantile Krabbe patients. Interestingly, the levels of LysoHexSph were reduced in the patient treated by HSCT, whereas those of C18-sulfatide remained high as in untreated patients. As for MEDNIK and MEDNIK-like syndromes, the mechanism(s) causing the elevation of C16-sulfatide still remains unknown, and further studies are needed to elucidate the relationship between adaptor protein machinery and sulfatide(s) metabolism.

## Conclusion

The presented method represents a technological innovation allowing the simultaneous diagnosis of a large number of NMD through a high-throughput multiplex LC-MS/MS analysis. The potential clinical applications include its use as first- or second-tier testing for extended NBS panels, as a diagnostic tool for the selective screening in patients with neurodegenerative diseases, as confirmatory testing for variants identified through whole exome sequencing or customized genetic panels (40) and for longitudinal biomarker monitoring in patients with treatable NMD.

## Data Availability

All pertinent data are presented explicitly in the text and supplemental data. Additional data regarding the characteristics of the participants (patients) and the levels of biomarkers in individual subjects may be available upon request.

## Supplemental Data

This article contains supplemental data.

## Conflict of Interest

The authors declare a potential conflict of interest as the analytical method described in this article has been licensed in a patent application for the analytical method (WO 2023/233433 A1, World Intellectual Property Organization).

## References

[1] Ferreira C.R., Rahman S., Keller M., Zschocke J., ICIMD Advisory Group (2021). An international classification of inherited metabolic disorders (ICIMD). J. Inherit. Metab. Dis..

[2] Parenti G., Medina D.L., Ballabio A. (2021). The rapidly evolving view of lysosomal storage diseases. EMBO Mol. Med..

[3] Kido J., Sugawara K., Nakamura K. (2023). Gene therapy for lysosomal storage diseases: current clinical trial prospects. Front. Genet..

[4] Saudubray J.M., García-Cazorla A., Saudubray J.M., R Baumgartner M., García-Cazorla A., Walter J. (2022). Inborn Metabolic Diseases, Diagnosis and Treatment.

[5] Almeida L.S., Pereira C., Aanicai R., Schröder S., Bochinski T., Kaune A. (2022). An integrated multiomic approach as an excellent tool for the diagnosis of metabolic diseases: our first 3720 patients. Eur. J. Hum. Genet..

[6] Pettazzoni M., Froissart R., Pagan C., Vanier M.T., Ruet S., Latour P. (2017). LC-MS/MS multiplex analysis of lysosphingolipids in plasma and amniotic fluid: a novel tool for the screening of sphingolipidoses and Niemann-Pick type C disease. PLoS One.

[7] Polo G., Burlina A.P., Ranieri E., Colucci F., Rubert L., Pascarella A. (2019). Plasma and dried blood spot lysosphingolipids for the diagnosis of different sphingolipidoses: a comparative study. Clin. Chem. Lab. Med..

[8] Hong X., Sadilek M., Gelb M.H. (2020). A highly multiplexed biochemical assay for analytes in dried blood spots: application to newborn screening and diagnosis of lysosomal storage disorders and other inborn errors of metabolism. Genet. Med..

[9] Polo G., Burlina A.P., Kolamunnage T.B., Zampieri M., Dionisi-Vici C., Strisciuglio P. (2017). Diagnosis of sphingolipidoses: a new simultaneous measurement of lysosphingolipids by LC-MS/MS. Clin. Chem. Lab. Med..

[10] Laugwitz L., Mechtler T.P., Janzen N., Oliva P., Kasper A.R., Teunissen C.E. (2024). Newborn screening and presymptomatic treatment of metachromatic leukodystrophy. N. Engl. J. Med..

[11] Ducatez F., Mauhin W., Ottaviani J., Plichet T., Pilon C., Lidove O. (2025). Lysosphingolipid quantitation in plasma and dried-blood spots using targeted high-resolution mass spectrometry. J. Clin. Lab. Anal..

[12] Gelb M.H., Basheeruddin K., Burlina A., Chen H.J., Chien Y.H., Dizikes G. (2022). Liquid chromatography–tandem mass spectrometry in newborn screening laboratories. Int. J. Neonatal. Screen..

[13] Malvagia S., Forni G., Ombrone D., La Marca G. (2020). Development of strategies to decrease false positive results in newborn screening. Int. J. Neonatal. Screen..

[14] Menkovic I., Boutin M., Alayoubi A., Curado F., Bauer P., Mercier F.E. (2022). Quantitation of a plasma biomarker profile for the early detection of Gaucher disease type 1 patients. Bioanalysis.

[15] Malvagia S., Ferri L., Della Bona M., Borsini W., Cirami C.L., Dervishi E. (2021). Multicenter evaluation of use of dried blood spot compared to conventional plasma in measurements of globotriaosylsphingosine (LysoGb3) concentration in 104 Fabry patients. Clin. Chem. Lab. Med..

[16] Natarajan A., Christopher R., Netravathi M., Bhat M., Chandra S.R. (2018). Liquid chromatography-tandem mass spectrometry method for estimation of a panel of lysophosphatidylcholines in dried blood spots for screening of X-linked adrenoleukodystrophy. Clin. Chim. Acta.

[17] Saville J.T., Smith N.J., Fletcher J.M., Fuller M. (2017). Quantification of plasma sulfatides by mass spectrometry: utility for metachromatic leukodystrophy. Anal. Chim. Acta.

[18] Spacil Z., Babu Kumar A., Liao H.C., Auray-Blais C., Stark S., Suhr T.R. (2016). Sulfatide analysis by mass spectrometry for screening of metachromatic leukodystrophy in dried blood and urine samples. Clin. Chem..

[19] Moyano A.L., Pituch K., Li G., van Breemen R., Mansson J.E., Givogri M.I. (2013). Levels of plasma sulfatides C18:0 and C24:1 correlate with disease status in relapsing-remitting multiple sclerosis. J. Neurochem..

[20] Mirzaian M., Kramer G., Poorthuis B.J. (2015). Quantification of sulfatides and lysosulfatides in tissues and body fluids by liquid chromatography-tandem mass spectrometry. J. Lipid Res..

[21] Bekri S., Bley A., Brown H.A., Chanson C., Church H.J., Gelb M.H. (2024). Higher precision, first tier newborn screening for metachromatic leukodystrophy using 16:1-OH-sulfatide. Mol. Genet. Metab..

[22] Hong X., Daiker J., Sadilek M., Ruiz-Schultz N., Kumar A.B., Norcross S. (2021). Toward newborn screening of metachromatic leukodystrophy: results from analysis of over 27,000 newborn dried blood spots. Genet. Med..

[23] Su P., Khaledi H., Waggoner C., Gelb M.H. (2021). Detection of GM1-gangliosidosis in newborn dried blood spots by enzyme activity and biomarker assays using tandem mass spectrometry. J. Inherit. Metab. Dis..

[24] Van Baelen A., Roosens L., Devos S., Verhulst S., Eyskens F. (2023). A new multiplex analysis of glucosylsphingosine and globotriaosylsphingosine in dried blood spots by tandem mass spectrometry. Mol. Genet. Metab. Rep..

[25] Mak J., Cowan T.M. (2021). Detecting lysosomal storage disorders by glycomic profiling using liquid chromatography mass spectrometry. Mol. Genet. Metab..

[26] García-Cazorla A., Oyarzábal A., Saudubray J.M., Martinelli D., Dionisi-Vici C. (2022). Genetic disorders of cellular trafficking. Trends Genet..

[27] Martinelli D., Travaglini L., Drouin C.A., Ceballos-Picot I., Rizza T., Bertini E. (2013). MEDNIK syndrome: a novel defect of copper metabolism treatable by zinc acetate therapy. Brain.

[28] Dionisi Vici C., Sidorina A., Catesini G., Rizzo C. (2023). https://patentscope.wipo.int/search/en/detail.jsf?docId=US465980146&amp;_cid=P12-MH710E-59705-55.

[29] Welford R.W.D., Farine H., Steiner M., Garzotti M., Dobrenis K., Sievers C. (2022). Plasma neurofilament light, glial fibrillary acidic protein and lysosphingolipid biomarkers for pharmacodynamics and disease monitoring of GM2 and GM1 gangliosidoses patients. Mol. Genet. Metab. Rep..

[30] Kuchar L., Sikora J., Gulinello M.E., Poupetova H., Lugowska A., Malinova V. (2017). Quantitation of plasmatic lysosphingomyelin and lysosphingomyelin-509 for differential screening of Niemann-Pick A/B and C diseases. Anal. Biochem..

[31] Voorink-Moret M., Goorden S.M.I., van Kuilenburg A.B.P., Wijburg F.A., Ghauharali-van der Vlugt J.M.M., Beers-Stet F.S. (2018). Rapid screening for lipid storage disorders using biochemical markers. Expert center data and review of the literature. Mol. Genet. Metab..

[32] Billington C.J., Rayannavar A., Tryon R., Kaye T., Gupta A., Lund T.C. (2025). Prognostication and biomarker potential of C26:0 lysophosphatidylcholine in adrenoleukodystrophy. JAMA Pediatr..

[33] Bonaventura E., Alberti L., Lucchi S., Cappelletti L., Fazzone S., Cattaneo E. (2023). Newborn screening for X-linked adrenoleukodystrophy in Italy: diagnostic algorithm and disease monitoring. Front. Neurol..

[34] Jaspers Y.R.J., Yska H.A.F., Bergner C.G., Dijkstra I.M.E., Huffnagel I.C., Voermans M.M.C. (2024). Lipidomic biomarkers in plasma correlate with disease severity in adrenoleukodystrophy. Commun. Med. (Lond.).

[35] Morales-Romero B., González de Aledo-Castillo J.M., Fernández Sierra C., Martínez Carreira C., Zaragoza Bonet C., Fernández Bonifacio R. (2024). Plasma C24:0- and C26:0-lysophosphatidylcholines are reliable biomarkers for the diagnosis of peroxisomal β-oxidation disorders. J. Lipid Res..

[36] Jaspers Y.R.J., Ferdinandusse S., Dijkstra I.M.E., Barendsen R.W., van Lenthe H., Kulik W. (2020). Comparison of the diagnostic performance of C26:0-Lysophosphatidylcholine and very long-chain fatty acids analysis for peroxisomal disorders. Front. Cell Dev. Biol..

[37] Haynes C.A., De Jesús V.R. (2012). Improved analysis of C26:0-lysophosphatidylcholine in dried-blood spots via negative ion mode HPLC-ESI-MS/MS for X-linked adrenoleukodystrophy newborn screening. Clin. Chim. Acta.

[38] Hubbard W.C., Moser A.B., Liu A.C., Jones R.O., Steinberg S.J., Lorey F. (2009). Newborn screening for X-linked adrenoleukodystrophy (X-ALD): validation of a combined liquid chromatography–tandem mass spectrometric (LC–MS/MS) method. Mol. Genet. Metab..

[39] Svennerholm L., Vanier M.T., Månsson J.E. (1980). Krabbe disease: a galactosylsphingosine (psychosine) lipidosis. J. Lipid Res..

[40] Al Khudari R., Baqla S., Al Asmar D. (2025). Diagnostic impact of whole exome sequencing in neurometabolic disorders in Syrian children: a single center experience. Orphanet J. Rare Dis..

