# Supplementary material for: New multiplex LC-MS/MS method for lipid biomarker analysis of inherited neurodegenerative metabolic diseases
Source: J Lipid Res. 2025 Dec 20;67(1):100967. doi: 10.1016/j.jlr.2025.100967 (PMC12856316; doi:10.1016/j.jlr.2025.100967)
Supplement: Supplementary Tables S1–S10 and Figures S–S3 — : [file mmc1.pdf]

SUPPLEMENTAL INFORMATION:

New Multiplex LC-MS/MS Method for lipid biomarker Analysis of Inherited Neurodegenerative Metabolic Diseases

Anna Sidorina[1], Giulio Catesini[1], Federica Deodato[1], Sara Boenzi[1], Diego Martinelli[1], Cristiano Rizzo[1], Carlo Dionisi-Vici[1]

[1]Division of Metabolic Diseases and Hepatology, Bambino Gesù Childrens Hospital IRCCS, Rome, Italy

Supplemental Table S1. Quality control plasma samples with added concentrations.

**Analyte**	**QC 1**	**QC 2**	**QC 3**
LysoGb3	endogenous	endogenous + 2 nmol/L	endogenous + 20 nmol/L
LysoGM1	endogenous	endogenous + 10 nmol/L	endogenous + 100 nmol/L
LysoGM2	endogenous	endogenous + 10 nmol/L	endogenous + 100 nmol/L
LysoHexSph	endogenous	endogenous + 10 nmol/L	endogenous + 100 nmol/L
LysoSM	endogenous	endogenous + 10 nmol/L	endogenous + 100 nmol/L
C18-sulfatide	endogenous	endogenous + 20 nmol/L	endogenous + 200 nmol/L
C16-sulfatide	endogenous	endogenous + 100 nmol/L	endogenous + 1000 nmol/L
LPC 26:0	endogenous	endogenous + 100 nmol/L	endogenous + 1000 nmol/L

Supplemental Table S2. Quality control DBS samples with added concentrations.

**Analyte**	**QC 1**	**QC 2**	**QC 3**
LysoGb3	endogenous	endogenous + 100 nmol/L	endogenous + 200 nmol/L
LysoGM1	endogenous	endogenous + 250 nmol/L	endogenous + 500 nmol/L
LysoGM2	endogenous	endogenous + 250 nmol/L	endogenous + 500 nmol/L
LysoHexSph	endogenous	endogenous + 250 nmol/L	endogenous + 500 nmol/L
LysoSM	endogenous	endogenous + 100 nmol/L	endogenous + 200 nmol/L
C18-sulfatide	endogenous	endogenous + 250 nmol/L	endogenous + 500 nmol/L
C16-sulfatide	endogenous	endogenous + 250 nmol/L	endogenous + 500 nmol/L
LPC 26:0	endogenous	endogenous + 250 nmol/L	endogenous + 500 nmol/L

Supplemental Table S3. LC gradient at 0.6µl/min flow.

Time (min)	Phase A	Phase B
0.00	90	10
1.00	90	10
2.00	80	20
3.30	15	85
3.31	10	90
4.00	10	90
5.00	10	90
5.01	90	10
5.40	90	10

Supplemental Table S4. List of MRM transitions and settings used in the polarity-switching MS/MS method.

**Positive ionization mode 5500 V**					
ID transition	Q1 (Da)	Q3 (Da)	DP (V)	CE (V)	CXP (V)
LysoGM1-1	1280.7	204.2	220	70	10
LysoGM1-2	1280.7	366.3	220	57	10
LysoGM1-3*	1280.7	989.5	220	50	10
LysoGB3	786.4	282.4	120	53	10
Lyso509	509.5	184.1	120	35	10
LysoSM	465.4	184.1	100	30	9
LysoHexSph	462.4	282.3	60	30	14
LysoSM-D7	472.4	184.2	100	30	9
LPC26:0	636.5	184.3	50	40	9
LPC26:0-1*	636.5	104.0	50	40	9
LysoGM2-1	1118.5	827.0	250	50	10
LysoGM2-2*	1118.5	282.3	250	60	10
LPC26:0-D4	640.9	184.0	50	40	9
**Negative ionization mode -4500 V**					
C18-sulfatide	806.4	97.0	-100	-150	-5
C16-sulfatide	778.5	97.0	-80	-140	-6
C18-D3-sulfatide	809.4	97.0	-100	-130	-5
C16:1-OH-sulfatide	792.5	97.0	-100	-120	-11
C16-OH-sulfatide	794.5	97.0	-100	-120	-11
DHCA	433.6	433.6	-170	-5	-11
THCA	449.5	449.5	-160	-5	-11
DHCA-D3	436.4	436.4	-170	-5	-11
THCA-D3	452.3	452.3	-170	-5	-11

*transition used for quantification.

DP-declustering potential; CE-collision energy; CXP- collision cell exit potential.

SupplementalTable S5. Precision and accuracy assay for plasma samples.

	QC 1			QC 2				QC 3			
	Conc (nM)	CV (%) interd.	CV (%) intrad.	Conc (nM)	CV (%) interd.	CV (%) intrad.	Recov. (%)	Conc (nM)	CV (%) interd.	CV (%) intrad.	Recov. (%)
LysoGM1	nd	nd	nd	10.0	4	1	100	95.7	5	4	96
LysoGM2	nd	nd	nd	9.5	9	8	95	97.3	9	8	97
LysoGB3	0.29	12	12	2.3	5	4	101	19.3	6	4	95
lysoSM	6.9	6	2	16.5	2	2	96	107	1	2	100
LysoHexSph	0.96	8	5	11.2	7	3	102	105	7	4	104
LPC26:0	419	15	11	540	9	4	121	1600	6	3	118
C18-sulfatide	18.7	8	2	40.2	6	3	108	237	7	4	109
C16-sulfatide	294	19	12	416	17	8	122	1500	12	10	121
Lyso509*	1.10	6	5	1.02	9	5	nd	1.10	11	3	nd
C16:1-OH-sulfatide*	0.80	6	4	0.86	6	5	nd	0.90	8	4	nd
C16-OH-sulfatide*	1.01	8	5	0.93	9	4	nd	0.94	9	5	nd
DHCA	4505	16	2	-	-	-	-	-	-	-	-
THCA	962	17	1.3	-	-	-	-	-	-	-	-

*calculated MOM

Supplemental Table S6. Precision and accuracy assay for DBS samples.

	QC 1			QC 2				QC 3			
	Conc (nM)	CV (%) interd.	CV (%) intrad.	Conc (nM)	CV (%) interd.	CV (%) intrad.	Recov. (%)	Conc (nM)	CV (%) interd.	CV (%) intrad.	Recov. (%)
LysoGM1	nd	nd	nd	281	18	15	112	578	13	13	116
LysoGM2	nd	nd	nd	219	27	28	88	426	18	24	85
LysoGB3	nd	nd	nd	105	13	10	105	176	10	6	88
LysoSM	12.5	23	16	96.2	5	3	84	181	9	6	84
LysoHexSph	12.2	13	9	270	7	4	103	462	12	2	90
LPC26:0	31.8	13	10	312	24	1	112	560	21	2	106
C18-sulfatide	12.6	16	8	298	3	2	114	502	3	3	98
C16-sulfatide	332	11	5	549	8	4	87	825	10	4	99
lyso509*	0.46	10	4	0.50	5	5	nd	0.60	7	4	nd
C16:1-OH-sulfatide*	2.08	6	4	1.96	8	3	nd	1.99	8	4	nd
C16-OH-sulfatide*	1.73	4	5	1.71	7	2	nd	1.61	5	3	nd
DHCA	nd	nd	nd	-	-	-	-	-	-	-	-
THCA	nd	nd	nd	-	-	-	-	-	-	-	-

*calculated MOM

Supplemental Table S7. Limits of detection (LOD) and quantification (LOQ).

	Plasma		DBS	
	LOD (nM)	LOQ (nM)	LOD (nM)	LOQ (nM)
LysoGM1	0.6	2.0	3.5	12
LysoGB3	0.2	0.4	0.7	2.1
LysoSM	0.2	0.6	0.5	1.5
LysoHexSph	0.3	1.0	2.5	8
LPC26:0	0.2	0.7	1.7	5
LysoGM2	2.7	8.25	60	125
C18-sulfatide	0.5	1.5	1.0	3.5
C16-sulfatide	10	30	10	31
DHCA	20	60	nd	nd
THCA	5	2	nd	nd

nd - not detectable.

Supplemental Table S8. Concentrations of biomarkers in plasma.

	Samples	LysoGM1 (nM)		LysoGM2 (nM)		LysoGB3 (nM)		LysoHexSph (nM)		LysoSM (nM)		Lyso509 MOM		LPC 26:0 (nM)		C18-sulfatide (nM)		C16-sulfatide (nM)		C16:1-OH-sulfatide MOM		C16-OH-sulfatide MOM	
	**n.**	**median**	**range**	**median**	**range**	**median**	**range**	**median**	**range**	**median**	**range**	**median**	**range**	**median**	**range**	**median**	**range**	**median**	**range**	**median**	**range**	**median**	**range**
**Controls (n.122)**	122	nd	-	nd	-	0.4	0.2 - 0.7	2.4	0.7 - 6.1	8.7	3.9 - 18.4	1.0	0.5 - 3.5	300	167 - 478	18.7	9.4-48	201	78 - 406	1.0	0.1 - 2.2	1.0	0.2 - 2.1
**GM1 gangliosidosis (n.9)**	9	1.6	0.6 - 93.5	nd	-	0.6	0.3-1.2	4.9	1.0 - 9.2	20.5	4.3-31.7	3.1	0.8 - 8.6	363	187 - 482	18.0	13.5 - 28.5	217	127 - 444	1.2	0.5 - 3.5	1.0	0.6 - 1.6
**GM2 gangliosidosis (n.4)**	4	nd	-	55.4	3.8 - 104	0.7	0.4 - 1.1	1.0	0.7 - 1.1	4.1	2.2 - 6.2	3.8	0.8 - 10.1	322	190-420	35.4	31.8-46	218	100 - 250	1.1	0.4 - 1.7	0.9	0.5 - 1.4
**GM2. Tay-Sachs (n.2)**	2	nd	-	53.8	3.8-104	0.5	0.4-0.5	1.0	1.0 - 1.1	4.8	3.5 - 6.2	5.5	0.8 - 10.1	322	256 - 389	40	33.3 - 46.0	218	196 - 241	1.1	0.7 - 1.5	1.0	0.6 - 1.4
**GM2. Sandhoff (n.2)**	2	nd	-	55.4	49.9-60.9	0.9	0.8-1.1	0.8	0.7 - 1.0	3.4	2.2 - 4.6	3.8	1.5-6.0	305	190-420	34.7	31.8-37.5	175	100-250	1.1	0.4-1.7	0.8	0.5-1.2
**Fabry male naïve (n.2)**	2	nd	-	nd	-	206	39.5-373	1.2	1.1-1.2	8.4	7.1-9.8	5.5	3.8-7.3	208	128-289	17.5	9.9-25.1	215	129-302	1.0	0.5-1.5	1.1	0.7-1.5
**Fabry female (n. 1)**	1	nd	-	nd	-	6.5	-	1.0	-	4.2	-	1.1	-	147	-	17.8	-	196	-	1.5	-	1.1	-
**Fabry female carrier (n.2)**	2	nd	-	nd	-	3.4	2.5-4.4	0.8	0.7-0.9	5.1	5.0-5.1	0.7	0.6-0.9	205	202-209	28.3	23.3-33.4	251	222-280	1.2	0.6-1.8	0.8	0.6-1.1
**Gaucher naïve, type 1 (n.1)**	1	nd	-	nd	-	2.3	-	317	-	47	-	12	-	296	-	12	-	200	-	1.0	-	1.1	-
**Gaucher naïve, type 2 (n.1)**	1	nd	-	nd	-	4.4	-	1263	-	6.2	-	4.6	-	324	-	11	-	40	-	0.3	-	0.2	-
**Inf. Krabbe, classical form naïve (n.4)**	4	nd	-	nd	-	0.3	0.2-0.6	39	28.9-41.1	4.7	3.6-7.6	1.3	0.7-3.3	355	213-407	71.0	56.1-91.0	197	137-258	0.7	0.4-2.2	0.6	0.2-3.0
**Inf. Krabbe, HSCT treated (n.1)**	5	nd	-	nd	-	0.5	0.3-0.7	10.3	9.8-11.7	4.4	2.4-9.2	1.2	0.3-1.4	228	135-402	74.4	41.5-102	430	145-463	3.2	0.7-4.9	2.3	0.6-3.5
**ASMD (n.6)**	7	nd	-	nd	-	0.4	0.3-0.7	1.3	0.7-1.8	809	63 - 2088	763	93-1281	284	201-415	18.1	10.8-41.1	172	124-310	0.9	0.5-1.2	0.8	0.6-1.4
**NPC (n.16)**	23	nd	-	nd	-	0.4	0.2-0.7	1.7	0.8-5.7	26.1	10.8-120	226	77-432	251	114-391	21.1	8.9-34.6	244	78-502	0.9	0.3-2.1	1.1	0.4-2.2
**LAL (n.4)**	5	nd	-	nd	-	0.4	0.3-0.7	3.1	1.8-4.8	13.8	8.0-25.1	21.2	8.4-89.1	503	258-580	30.2	10.6-37.5	202	58.5-331	1.8	0.2-2.0	1.2	0.2-2.1
**X-ALD, male (n.8)**	8	nd	-	nd	-	0.5	0.3-0.8	1.2	1.1-1.5	9.3	7.9 - 16.5	1.5	0.9-3.2	2980	2013-4248	14.9	9.3-23.9	227	143-358	1.0	0.6-1.1	1.2	0.8-1.6
**AMN, male (n.11)**	11	nd	-	nd	-	0.6	0.4-1.0	1.9	1.1-2.7	16.7	9.1-20.1	2.4	1.4-5.5	2788	1253-6548	14.1	10.2-22.1	264	168-583	1.6	1.0-3.0	1.3	1.0-2.6

**X-ALD, female carrier (n.5)**	5	nd	-	nd	-	0.6	0.4-0.9	1.4	1.3-2.5	13.2	9.1-18.1	2.3	1.1-2.9	2433	1463-3433	18.8	15.9-22.1	250	202-380	1.5	1.4-2.9	1.6	1.0-2.3
**PBD (n.6)**	6	nd	-	nd	-	0.4	0.2-0.5	1.1	1.0-1.8	7.4	5.8-13.2	2.1	0.8-3.0	8573	6098-19098	17.0	10.7-49.8	175	81.3-273	0.8	0.3-1.4	0.9	0.4-1.4
**MLD, late infantile (n.1)**	2	nd	-	nd	-	0.4	0.4-0.5	1.2	1.1-1.3	9.6	9.1-10.0	2.3	1.1-3.5	520	420-621	335	285-385	1060	993-1127	19.4	19.1-19.7	7.2	6.8-7.5
**MLD, juvenile (n.3)**	4	nd	-	nd	-	0.4	0.3-0.5	0.8	0.5-1.1	8.2	5.1-12.6	0.8	0.6-1.4	358	196-558	37.5	23.8-68.5	342	273-504	5.5	4.5-9.0	2.4	1.9-3.4
**MEDNIK, MEDNIK-like (n. 1, 3)**	4	nd	-	nd	-	0.4	0.2-0.5	1.5	0.9-1.9	7.4	3.8-8.3	1.4	1.0-5.0	416	192-683	33.4	28.5-73.6	1434	1139-3537	1.4	0.9-2.5	1.1	0.4-2.2

nd - not detectable.

Supplemental Table S9. Concentrations of biomarkers in DBS.

	Samples	LysoGM1 (nM)		LysoGM2 (nM)		LysoGB3 (nM)		LysoHexSph (nM)		LysoSM (nM)		Lyso509 MOM		LPC 26:0 (nM)		C18-sulfatide (nM)		C16-sulfatide (nM)		C16:1-OH-sulfatideMOM		C16-OH-sulfatide MOM	
	**n.**	**median**	**range**	**median**	**range**	**median**	**range**	**median**	**range**	**median**	**range**	**median**	**range**	**median**	**range**	**median**	**range**	**median**	**range**	**median**	**range**	**median**	**range**
**Controls (n.188)**	188	nd	-	nd	-	0.9	<0.7-1.6	4.0	<2.5-13.6	43.4	18.6-84.5	1.0	0.4-2.8	28.6	14.6-92.7	14.3	7.0-29.4	198	83.5-482	1.2	0.4-2.2	1.0	0.3-2.0
**GM1 gangliosidosis (n.5)**	5	nd	nd-8.0	nd	-	nd	<0.7	2.5	1.0-5.3	47.4	23.6-76.6	2.7	1.8-8.7	35.6	29.8-57.9	12.7	10.2-15.4	181	99-293	1.5	0.3-4.6	1.1	0.4-2.1
**GM2 gangliosidosis (n.4)**	4	nd	-	nd	-	nd	<0.7-1.3	4.1	<2.5-10.7	76.4	43.7-85.4	2.1	1.3-2.7	33.5	32.4-39.7	19.8	15.6-24.8	120	107-166	0.9	0.6-1.5	1.0	0.5-1.2
**Fabry male naïve, (n.1)**	1	nd	-	nd	-	84.7	-	1.2	-	51.0	-	2.1	-	41.4	-	11.9	-	149	-	1.5	-	1.4	-
**Fabry female (n. 1)**	1	nd	-	nd	-	8.9	-	1.5	-	51.4	-	1.9	-	36.9	-	13.7	-	148	-	2.1	-	1.7	-
**Fabry female carrier (n.2)**	2	nd	-	nd	-	6.7	6.4-6.9	2.0	1.3-2.7	24.6	3.4-45	1.1	0.3-1.8	32.9	23.1-42.6	17.3	13.7-21.0	219	175-263	1.9	1.4-2.3	1.9	1.4-2.5
**Gaucher naïve, type 1 (n.1)**	1	nd	-	nd	-	nd	-	160.5	-	95.0	-	1.9	-	45.4	-	10.8	-	205	-	1.9	-	1.9	-
**Inf. Krabbe, classical form naïve (n.1)**	1	nd	-	nd	-	nd	-	19.6	-	60.6	-	2.0	-	39.0	-	45.4	-	131	-	1.6	-	1.0	-
**Inf. Krabbe, HSCT treated (n.1)**	1	nd	-	nd	-	nd	-	7.5	-	62.6	-	4.1	-	40.4	-	35.6	-	260	-	2.8	-	1.8	-
**ASMD (n.2)**	2	nd	-	nd	-	nd	-	2.9	1.9-4.0	942	652-1232	30.3	27.7-33.0	72.4	51.6-93.1	12.9	9.6-16.1	99.1	98.1-100	0.8	0.4-1.2	0.5	0.3-0.8
**NPC (n.7)**	11	nd	-	nd	-	0.75	<0.7-0.77	1.8	1.2-8.9	54.2	9.6-99.4	6.6	3.7-12.7	31.3	14.3-54.6	12.3	6.2-14.6	155	105-218	1.0	0.5-1.6	1.6	0.7-2.0
**LAL (n.3)**	3	nd	-	nd	-	1.5	<0.7-1.5	2.5	1.4-2.5	48.6	35.8-61.0	1.7	1.3-6.6	33.2	16.4-36.4	13.2	11.6-13.8	157	110-168	1.9	1.5-2.0	1.7	1.2-1.9
**X-ALD, male (n.8)**	8	nd	-	nd	-	1.1	<0.7-1.1	4.5	1.2-9.7	10.2	6.1-16.5	1.0	0.2-1.9	173	134-522	8.3	4.5-15.8	122	82.4-208	1.0	0.6-1.6	0.8	0.5-1.3
**PBD (n.5)**	6	nd	-	nd	-	1.2	<0.7-1.2	2.7	1.3-7.9	28.8	6.9-42.6	1.5	0.4-7.2	480	296-788	14.2	8.0-17.8	201	112-256	2.0	1.2-2.6	1.8	1.4-3.3
**MLD, late infantile (n.1)**	2	nd	-	nd	-	0.8	<0.7-0.8	1.3	1.1-1.5	65.2	61.4-69.0	2.4	2.3-2.5	33.5	32.8-34.3	165	143-187	648	623-673	24.4	20.4-28.3	9.9	9.5-10.2
**MLD, juvenile (n.3)**	4	nd	-	nd	-	nd	-	1.5	0.6-1.6	48.8	27.2-69.4	1.9	1.2-6.3	24.9	22.1-39.2	16.6	10.8-28.8	350	237-535	8.5	4.7-10.8	4.2	2.3-5.1
**MEDNIK, MEDNIK-like (n. 1, 3)**	4	nd	-	nd	-	nd	-	2.8	0.8-4.3	38.8	4.3-110	1.4	0.1-3.2	34.6	17.2-43.0	16.9	13.7-32.1	865	672-1562	1.3	0.8-2.7	0.8	0.6-2.4

Supplemental Table S10. Concentrations of bile acids in plasma.

	n	Age	DHCA		THCA	
			median	range	median	range
**Controls**	122	0.1 – 63.3	nd	nd	nd	nd
**GM1 gangliosidosis**	6	0.7 -19.8	nd	nd	nd	nd
**GM2 gangliosidosis**	4	1.8- 3.1	nd	nd	nd	nd
**Tay-Sachs**	2	2.6-3.1	nd	nd	nd	nd
**Sandhoff**	2	1.8-2.1	nd	nd	nd	nd
**Fabry - female**	3	2.2-10.9	nd	nd	nd	nd
**Fabry - male**	2	9.7-10.1	nd	nd	nd	nd
**Krabbe - classical form**	4	0.1-0.9	nd	nd	nd	nd
**Krabbe - post transplant**	5	0.8-4.1	nd	nd	nd	nd
**Gaucher - naÏve**	2	0.1-15.3	nd	nd	nd	nd
**ASMD**	7	8.1-17.8	nd	nd	nd	nd
**NPC**	23	0.3-28.2	nd	nd	nd	nd
**ALD**	8	4.2-28.9	nd	nd	nd	nd
**AMN**	11	34.7-63.2	nd	nd	nd	nd
**ALD carrier**	5	36.7-71.8	nd	nd	nd	nd
**PBD**	6	0.1-20.4	580	61-5450	24	5.5-9450
**MEDNIK, MEDNIK-like**	4	1.2-15.8	nd	nd	nd	nd
**LAL**	5	0.5-14.8	nd	nd	nd	nd
**MLD - late infantile**	**2**	**2.8-3.1**	**nd**	**nd**	**nd**	**nd**
**MLD - juvenile**	**4**	**5.8-13.2**	**nd**	**nd**	**nd**	**nd**

nd - not detectable.

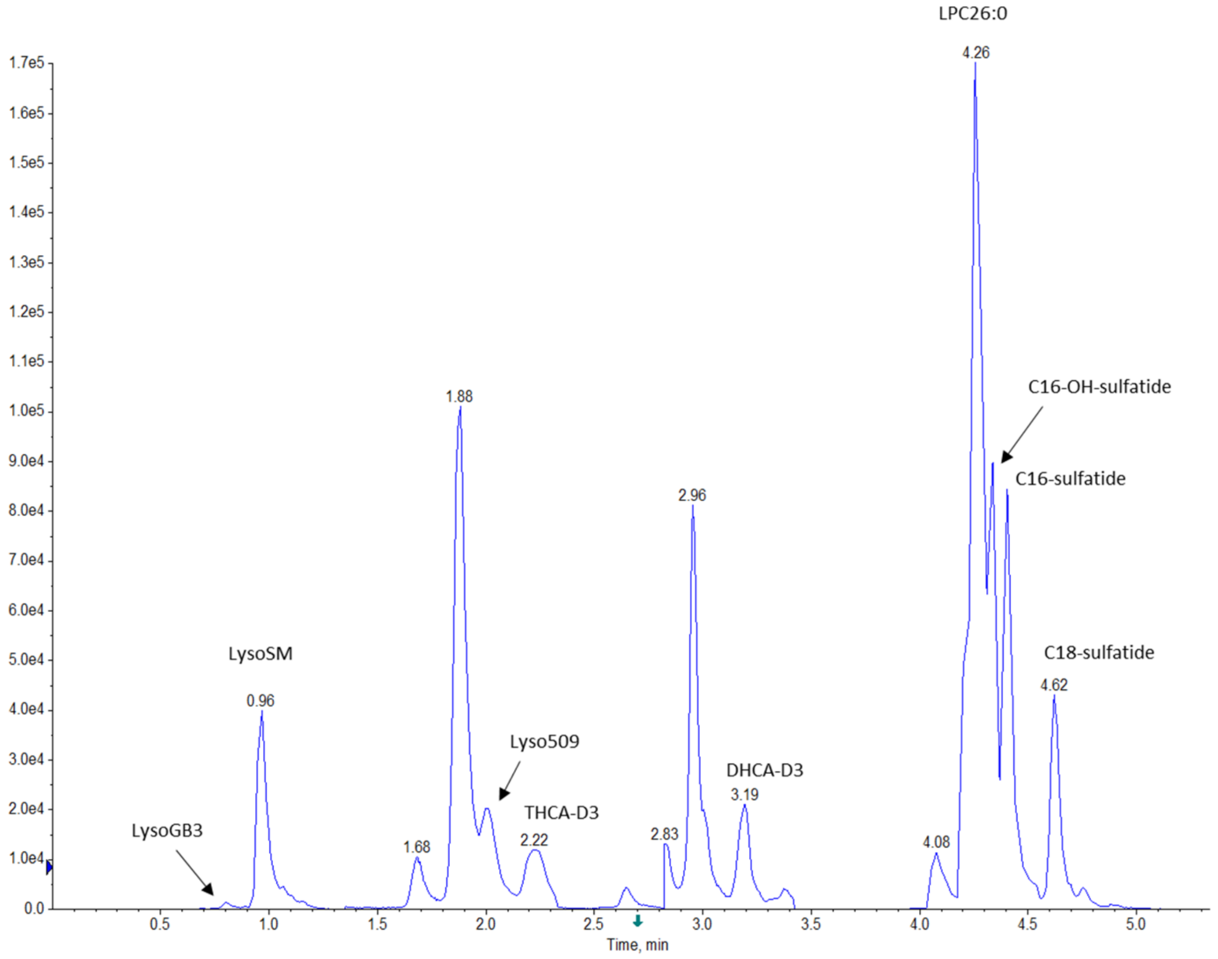

Supplemental Figure S1. The total ion current (TIC) acquired by switching polarity in positive and negative mode from a plasma sample.

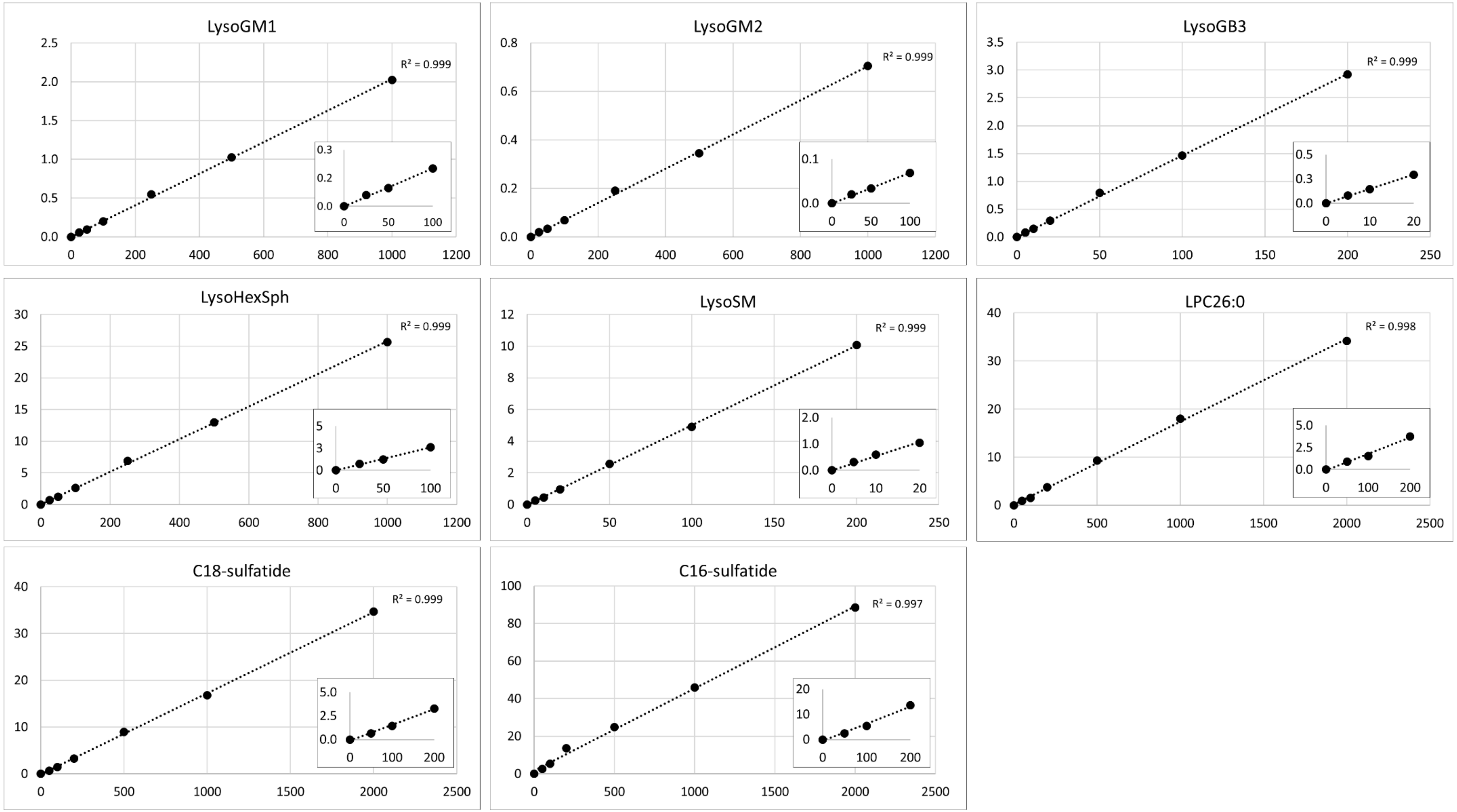

Supplemental Figure S2. Calibration curves of quantitatively assessed analytes in plasma.

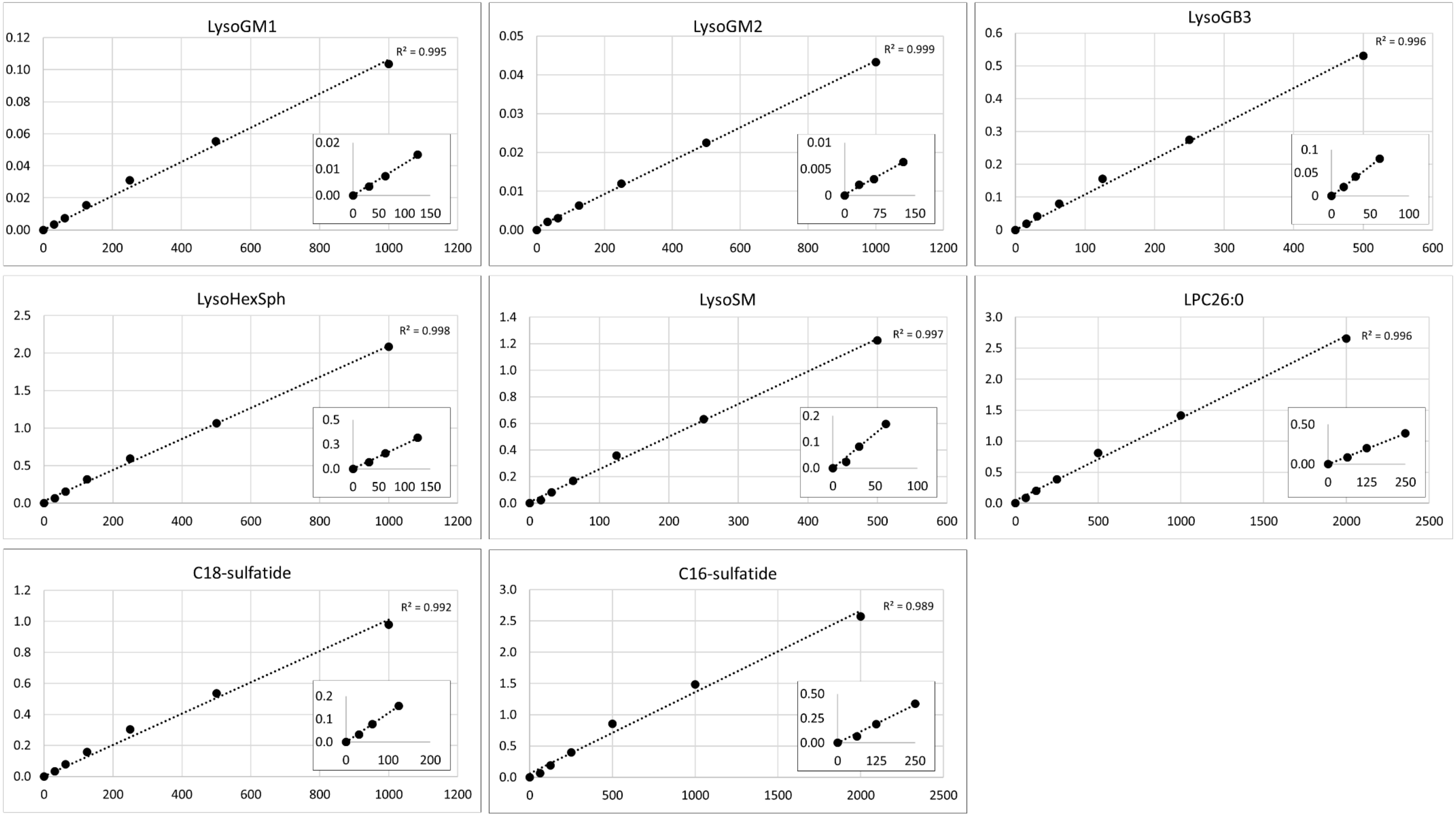

Supplemental Figure S3. Calibration curves of quantitatively assessed analytes in DBS.
